# MicroRNA Expression Profiles and Breast Cancer Chemotherapy

**DOI:** 10.3390/ijms221910812

**Published:** 2021-10-06

**Authors:** Matthew G. Davey, Aoife J. Lowery, Nicola Miller, Michael J. Kerin

**Affiliations:** Department of Surgery, The Lambe Institute for Translational Research, National University of Ireland, Galway, H91 YR71 Galway, Ireland; aoife.lowery@nuigalway.ie (A.J.L.); nicola.miller@nuigalway.ie (N.M.); michael.kerin@nuigalway.ie (M.J.K.)

**Keywords:** breast cancer, chemotherapy, precision oncology, personalised medicine, miRNA

## Abstract

Breast cancer is the most common malignancy diagnosed in women. Traditionally, radical surgical resection was the cornerstone of breast cancer management, with limited exceptions. In recent times, our enhanced appreciation of the biomolecular characteristics of breast cancer has transformed the treatment paradigm to include prescription of chemotherapeutical agents, radiotherapies, targeted therapies, as well as more refined surgical approaches. While treatments with such modalities have enhanced clinico-oncological outcomes for breast cancer patients, the efforts of oncological and translational research have concentrated on the identification of novel biomarkers which may successfully inform prognosis and response to therapies, improve current therapeutic strategies, and enhance prognostication. Mi(cro)RNAs are small, non-coding molecules which are known to play regulatory roles in governing gene expression and cellular activity. Measurement of miRNA expression profiles have been illustrated to inform the response to therapies, such as conventional chemotherapy, and are currently undergoing assessment as means of enhancing treatment strategies with these cytotoxic agents. Herein, this review outlines how chemotherapy prescription has revolutionised breast cancer treatment and illustrates the novel role of miRNAs as biomarkers capable of enhancing current therapeutic strategies using chemotherapy in patients being treated with curative intent for breast cancer.

## 1. Introduction

Breast cancer remains the most common malignancy in women, with estimations suggesting almost 1.7 million women are diagnosed and treated for breast cancer each year, contributing 11.9% to the global cancer burden [1]. Moreover, breast cancer accounts for 30% of all female cancers and 15–20% of all female cancer deaths [2]. Although there is an increasing incidence in breast cancer diagnoses in recent years [3], the prognosis of the disease has improved dramatically, with anticipated 5-year survival outcomes improving from 40% to almost 90% over the past 50 years [4]. Traditionally, en-bloc radical resections in the form of Halstead mastectomy and axillary clearance were believed to be fundamental in controlling breast cancer, with limited exceptions [5]. In more recent times, enhanced clinical outcomes have evolved in accordance with our increased appreciation of the molecular mechanisms underpinning the heterogeneity of breast tumours, which has facilitated more conservative surgery and the personalisation of treatment strategies to increase toxicity to the tumour while minimising unnecessary morbidity to the patient. This encompasses the era of precision oncology, which has identified breast cancer as a heterogeneous disease, leading to routine substratification of these cancers into four biological distinct, intrinsic molecular subtypes, all of which have varying clinical behaviour, prognoses, treatment strategies, as well as response rates to such treatments (i.e., luminal A breast cancer (LABC), luminal B breast cancer (LBBC), human epidermal growth factor receptor-2 enriched breast cancer (HER2+) and triple-negative breast cancer (TNBC) [6]. Currently, the St. Gallen expert consensus statements highlight gene expression profile (GEP) assays (e.g., Prosogna©—PAM50 messenger RNA (mRNA) expression signature, NanoString Technologies, Seattle, WA, USA; MammaPrint©, Agilent Technologies, CA, USA; or OncotypeDX Recurrence Score© (RS), Genomic Health Inc., Redwood City, CA, USA) as the gold standard for the substratification of luminal breast tumours into their distinct intrinsic biological subtypes, while routine appraisal of estrogen (ER), progesterone (PgR) and human epidermal growth factor receptor-2 (HER2) receptors, as well as Ki-67 proliferation indices using immunohistochemistry staining, remain critical in identifying the molecular subtypes in common clinical practice [7,8,9,10]. Routine assessment of such biomarkers remains fundamental in guiding therapeutic decision making regarding adjuvant chemoendocrine agents and targeted therapies. Nonetheless, the paradigm appears to be shifting towards the adoption of the aforementioned GEP signatures to modify treatment strategies suitable to each patient while providing sensitive prognostication and predicting response to therapies, therefore validating their inclusion in oncological guidelines (such as the American Society of Clinical Oncology (ASCO), European Society of Medical Oncology (ESMO), and National Institute for Health and Clinical Excellent (NICE) treatment guidelines) [11,12,13]. However, small, non-coding ribonucleic acids (RNA) have also been acknowledged to have value in personalised medicine.

Micro ribonucleic acids (microRNA or miRNA) are small, non-coding ribonucleic acids (RNA) which are key in regulating gene expression [14]. First described by Lee et al. in 1993 [15], miRNAs have a key role in cancer proliferation, with the clinical utility of prognostic, diagnostic, and therapeutic avenues being explored through measuring miRNA expression profiles [16]. As miRNAs are modulators of oncogenesis in breast carcinoma, assessing their utility in enhancing current chemotherapeutic strategies may be useful in improving the current treatment paradigm. Accordingly, the aim of the current review is to outline how breast cancer patient management has evolved such that robust chemotherapy prescriptions have enhanced clinico-oncological outcomes and to determine the potential role of microRNA in enhancing treatment with curative intent using chemotherapy in patients diagnosed with breast cancer.

## 2. Breast Cancer Chemotherapy

### 2.1. Adjuvant Chemotherapy

Complete surgical resection has traditionally provided effective breast cancer disease control [17]. William Halstead’s radical mastectomy (which involved extensive resection of all the breast parenchyma, locoregional lymph nodes, and pectoralis major muscle) was once considered the mainstay of breast cancer management, irrespective of disease burden [5,18]. The first chemotherapeutical regimen prescribed with curative intent in breast cancer was cyclophosphamide, methotrexate, and 5-fluorouracil (CMF) prescribed by Bonadanno et al. in 1976, which significantly reduced breast cancer recurrence (94.7% of 207 patients treated with chemotherapy versus 76.0% of 179 patients spared chemotherapy) [19]. Since the late 1950s, Bernard Fisher and his National Surgical Adjuvant Breast and Bowel Project (NSABP) hypothesised the limited scientific and biomolecular rationale for radical surgery in breast cancer, as this alone was often insufficient to establish total disease control [20]. The NSABP is a clinical trial cooperative group funded by the National Cancer Institute which is responsible for several landmark studies in the fields of breast and colonic oncology, including data supporting the added value of chemotherapy in cases of breast carcinoma [21]. Within the context of ER-/lymph-node-negative (LN-) disease, the NSABP-B13, B-19, and B-23 trials highlighted the non-inferiority of prescribing four cycles of doxorubicin and cyclophosphamide (AC) versus six cycles of CMF chemotherapeutical agents or methotrexate and 5-fluorouracil (MF), while also highlighting no benefit of adding tamoxifen (a selective estrogen receptor modulator) for this disease subtype [22,23,24]. Additionally, in their NSABP B-14 and B-20 trials, Fisher et al. established that within ER+ early disease, tamoxifen combined with chemotherapy (6-cycles of CMF and tamoxifen (CMFT) or methotrexate and 5-fluorouracil and leucovorin (MTF)) provided survival advantaged over tamoxifen alone (5-year disease-free survival (DFS): MTF 90%, CMFT 89%, tamoxifen alone 85%) while also demonstrating that tamoxifen provides enhanced 5-year, 10-year, and 15-year survival over placebo (5-year: 69% vs. 57%, *p* < 0.0001) [25,26,27]. Following the success of these clinical trials, the hypothesis evolved to identify those with ER+/LN− disease who derive benefit from combined adjuvant chemoendocrine therapy versus those who may be spared chemotherapy and treated with tamoxifen alone; through assessment of the resected specimen paraffin-embedded blocks from the NSABP B-14 and B-20 trials, Genomic Health Inc. (Redwood City, CA, USA) designed and validated a reverse transcriptase-polymerase chain reaction (RT-PCR) 21-gene assay (OncotypeDX Recurrence Score©) capable of predicting recurrence risk and estimating benefit from cytotoxic chemotherapy prescription in these patients [9]. The subsequent results of the TAILORx trial illustrated no survival advantage for post-menopausal patients, with RS < 25, implicating indication for endocrine therapy alone for these patients [28]. In more recent times, the expansion of indications into the locally advanced and neoadjuvant settings is likely based on preliminary data from the RxPONDER trial (recruiting patients with 1–3 positive nodes) and several meta-analyses [29,30,31,32]. The landmark clinical studies assessing the role of multigene expression assays for guiding adjuvant chemotherapy prescription in ER+ breast cancer are outlined in Supplementary Table S1. In summary, Fisher’s hypothesis that all breast cancer patients required systemic treatment (particularly with chemotherapy) has been successfully challenged. The molecular era allows us to personalise approaches to optimise outcomes for patients, minimise toxicity, and achieve disease control with less aggressive and more targeted therapies. The future will allow us to address specific markers of response to facilitate tumour eradication and limit the need for prolonged and excessive therapies. The scientific community is now addressing the value of measuring mi(cro)RNA expression (both tumour and circulating) which can potentially allow prescription of appropriate targeted treatments, address early relapse, and even allow specific miRNA directed therapies.

In the 1990s, the cytotoxic effect of taxane-based chemotherapy provided the rationale for their addition for evaluation in clinical trials in the adjuvant setting; the NSABP B-28 tial randomised patients to receive four cycles of paclitaxel following four cycles of conventional adriamycin and cyclophosphamide (AC) in patients with LN+ breast cancer, reducing the relative risk of DFS events by 17% (5-year DFS: 76% vs. 72%, *p* = 0.006) [33]. The NSABP B-30 trial then illustrated the enhanced survival in patients with LN+ disease who received four cycles of adriamycin and cyclophosphamide, followed by four cycles of docetaxel (ACT), versus upfront ACT or doxorubicin and docetaxel (8-year DFS: 74% vs. 69% vs. 69%, respectively). At this time, cancer researcher Axel Ullrich and medical oncologist Denis Slamon highlighted the substantial information accumulating in relation to the role of the HER2/neu oncogene in 20–25% of breast tumours and recognised targeting this receptor on the cell surface by a monoclonal antibody, trastuzumab, which could inhibit cell signaling, impair oncogenesis, and improve clinical outcomes for patients [34,35,36]. While trastuzumab was initially validated for use in metastatic HER2+ disease [37,38,39], results from landmark trials such as the HERA, NSABP B-31, and PHARE have seen the expansion of clinical indications for the prescription of trastuzumab into the adjuvant setting [40,41,42]. Randomised clinical trials outlining the role of trastuzumab in HER2+ disease are outlined in Table 1.

### 2.2. Neoadjuvant Chemotherapy

Oncological practice has evolved recognising the inherent value of treating patients with chemotherapy in the neoadjuvant setting. Advantages of neoadjuvant chemotherapy (NAC) included tumour downstaging, increasing patient eligibility for breast conservation surgery (BCS), as well as the generation of in vivo data in relation to tumour sensitivity, which has been illustrated to carry prognostic significance for disease recurrence and overall survival (OS) [52,53,54]. While DFS and OS outcomes are similar to those treated in the adjuvant setting, recent data from a meta-analysis of randomised trials conducted by the Early Breast Cancer Triallist’s Collaborative Group (EBCTCG) indicate that there are increased rates of locoregional recurrence (LRR) following neoadjuvant therapy (21.4% vs. 15.9%) [55]. Furthermore, there is increasing evidence outlining a survival advantage for those who achieve a pathological complete response (pCR) following NAC, compared with their contemporaries with residual disease [54,56].

In 1998, the seminal NSABP B-18 trial identified the benefit of NAC in their analysis of 1523 randomised patients with early breast cancer; 13% achieved a pCR (defined as absence of invasive tumour in the breast specimen following NAC), 36% achieved a complete clinical response, 43% achieved a partial clinical response, 37% of patients underwent axillary downstaging having presented with palpable LNs, and patients in receipt of NAC were more likely to undergo successful BCS (67% vs. 60%, *p* = 0.002) [57,58]. Moreover, there was no significant difference in DFS, distant DFS, and OS observed between both groups, although a non-significant difference in LRR was observed in those treated with NAC (10.7% vs. 7.6%, *p* = 0.120) [59]. In 2003, the NSABP B-27 randomised trial evaluated the role of adding four cycles of docetaxel to four cycles of neoadjuvant AC [60] and demonstrated increased pCR rates and axillary downstaging with added neoadjuvant docetaxel while failing to increase BCS rates, DFS, and OS outcomes [61].

Traditionally, histopathological features such as tumour size and degree of nodal involvement were the predominant selection criteria for NAC. However, the paradigm has evolved such that intrinsic tumour biology informs response rates to neoadjuvant therapies and predicts those likely to achieve pCR [62]. While molecular subtyping from diagnostic core biopsy remains critical in contemporary breast cancer management in relation to the indication for NAC, multigene expression assays (such as the 21-gene expression signature) are likely to indicate response to neoadjuvant therapies in early stage ER+ disease [29,30] (Supplementary Figure S1). With respect to HER2+ and TNBC, the clinical utility of NAC has become embedded into best-practice guidelines: A recent update from ASCO recommends the use of NAC and trastuzumab for HER2+ cancers (with the exception of T1a-T1b N0 disease), with anthracycline and taxane-based chemotherapy and trastuzumab to be utilised in high-risk LN- cases and those with LN positivity [63]. Furthermore, patients with TNBC should be offered an anthracycline and taxane-based regimen in all cases, unless diagnosed with cancer staged T1a-T1b N0 [63]. Based on the work of a recent meta-analysis, ASCO also endorse the addition of platinum-based chemotherapy in TNBC due to an increased propensity to achieve pCR (52.1% versus 37.0%) [64]. Preliminary results of the KEYNOTE522 trial supports anti-programmed death ligand-1 (PD-L1) inhibition through immune-checkpoint modulation (pembrolizumab) into practice to further enhance pCR rates (pembrolizumab and NAC: 64.8% versus placebo and NAC: 51.2%) [65]; however, ASCO report insufficient evidence at present for their inclusion in conventional neoadjuvant treatment for TNBC. Future directions for translational research efforts are focused on the extrapolation of enhancing pCR rates, facilitating the de-escalation of adjuvant treatment following pCR and reducing treatment-related toxicities for patients in receipt of these neoadjuvant therapies [66,67]. There is a vogue in recent times to suggest manipulation of treatment using miRNA replacement therapies may be useful in augmenting pCR rates to NAC in breast cancer, which is outlined in detail in this review. Table 2 outlines seminal studies validating NAC prescription in early breast cancer.

## 3. MicroRNAs

MiRNAs are a contemporary class of small (19–25 nucleotides in length) non-coding endogenous RNAs which are known to play key modulatory roles in gene expression and cellular processes [14]. They were first described by Lee et al. in 1993 when studying developmental timing of *Caenorhabditis elegans* [15]; scientific understanding of the role of miRNA has exponentially grown in recent years, with aberrant miRNA expression profiles now understood to correlate with several diverse pathological processes, including oncogenesis [70,71]. MiRNAs regulate gene expression at a post-transcriptional level by binding to the 3′ or 5′untranslated regions of target mRNA, hindering mRNA expression through degradation or translation inhibition [72]. Overall, miRNAs can be oncogenic (oncomirs) or tumour suppressors (tumour suppressor miRNA) and influence cancer development through each of these means.

The biogenesis of miRNA is a complex, multi-step process occurring initially in the cellular nucleus, before completing the production process in the cytoplasm: MiRNA genes are transcribed by RNA polymerase II/III in the nucleus to form large, capped, and polyadenylated primary miRNA transcripts (pri-miRNA) [73]. Next, pri-miRNAs are cleaved into pre-miRNA (which are 70–90 nucleotides in length) by the coupled RNase III enzyme Drosha and its complementary binding partner DCGR8. These pre-miRNAs are the precursor molecules to miRNAs and then exported out of the nucleus in their imperfect hairpin structure by the export protein (Exportin 5) [74]. These pre-miRNAs then undergo cleavage into double-stranded miRNAs in the cytoplasm by RNase type III Dicer with either the trans-activating RNA-binding protein (TRBP) or the protein activator of the interferon-induced protein kinase (PACT) [75], with one of these strands representing the mature miRNA which forms the RNA silencing complex in conjunction with several other proteins [76]. This mature strand is then incorporated into the miRNA-associated RNA-induced silencing complex (miRISC), which guides the RISC to target mRNA due to its complementary sequences to the mature mRNA, consequentially impacting on and altering gene expression. Several studies correlate miRNA expression profiles to important biopathologic and molecular subtyping data [77,78]; using a stepwise artificial neural network model in 95 tumours, Lowery et al. identified miRNA signatures capable of predicting ER, PgR, and HER2 receptors, indicating the crucial role of individual miRNA in deriving intrinsic biological breast cancer subtypes. Furthermore, Sokilde et al. validated the hypothesis that miRNA profiles largely recapitulate molecular subtypes [77,78]. Although we are now well acquainted with the various tumour suppressor/oncogenic roles of miRNA in cancer development, the aforementioned studies underpin the critical role of various miRNA such processes, with variations even observed among differing intrinsic biological subtypes of the disease. While these studies provide promise for the identification of novel molecular subtypes capable of being targeted with future therapeutic strategies to enhance oncological outcome, other authors focus on the current breast cancer treatment paradigm. These authors highlight the potential for miRNA signatures as predictive and prognostic biomarkers that could personalise breast cancer therapeutics and improve patient selection strategies for current therapies, such as conventional cytotoxic chemotherapies [79,80].

## 4. MicroRNAs in Predicting Response to Neoadjuvant Chemotherapies

As previously outlined, breast oncology has evolved in recent years to recognise it is strategic and advantageous to treat patients with chemotherapy in the neoadjuvant setting [57,58]. While conventional clinicopathological characteristics have been reported to correlate with response to NAC [53,81,82,83], deciphering those likely to achieve such responses remains challenging to the oncologists, with response rates often difficult to predict. Several recent studies correlate miRNA expression profiles with response to NAC for breast cancer: Xing et al. reported that increased expression of miR-23a-3p, miR-200c-3p, miR-214-3p and reduced expression of miR-451a and miR-638 correlated to chemoresistance (Miller–Payne grade 1) [84]. In the Clinical Trials Ireland All-Ireland Cooperative Oncology Research Group (CTRIAL-IE ICORG) 10/11 prospective, multicentre translational trial, McGuire et al. highlight the inherent value of miR-21 expression as a correlate to response to standard NAC in their analysis of 114 breast cancer patients [79]. Moreover, Liu et al. illustrate reduced miR-21 expression levels after cycle 2 of NAC in responders (versus non-responders), supporting the work of CTRIAL-IE ICORG 10/11 trial [85]. In the translation research arm of the NeoALTTO prospective study, Di Cosimo et al. outlined the clinical utility of venous sampling for miR-140a-5p, miR-148a-3p, and 374a-5p, and their predictive value in determining response to following neoadjuvant therapies [86], with an increased combined predictive capability of 54% in determining pCR to trastuzumab in HER2+ disease, compared with 0% in cases of poor expression. In the GeparSixto trial, Stevic et al. described how aberrant expression of miR-199a in patient plasma was predictive of pCR to NAC in their series of 435 patients diagnosed with either early stage HER2+ or TNBC disease [87]. Kassem et al. provided promising data supporting miR-34a expression levels to accurately discriminate responders from non-responders in 39 patients being treated for locally advanced breast cancer (area under the curve (AUC): 0.995, sensitivity: 97.4%, specificity: 100.0%) [88]. Garcia-Garcia reported reduced miR-145-5p expression levels in patients successfully achieving a pCR to NAC (AUC: 0.790, *p* = 0.003) [89]. Table 3 illustrates prospective trials evaluating the role of miRNAs in predicting response to neoadjuvant therapies and describing the miRNAs that are relevant in this settling. With respect to adjuvant chemotherapy, using miRNA expression profiles to measure response is significantly more challenging. Factors such as timing of miRNA sampling, crude assessment of response rates to treatment and quantifying whether therapy enhanced oncological outcomes for those likely to succumb to recurrence is extremely difficult. Thus, it is unsurprising that most studies measure miRNA expression profiles with metrics indication response (i.e.: RECIST, Miller–Payne grade, Sataloff score, etc.) to NAC and not adjuvant chemotherapy.

## 5. MicroRNAs and Chemoresistance

It has been well established that miRNAs are capable of increasing the resistance of cancer cells to conventional chemotherapeutic drugs, endocrine hormonal agents, and radiotherapies [97,98,99,100]. Regarding chemotherapeutic resistance, several reports have revealed scientific mechanisms and rationale for resistance, including alterations of drug-target interactions, reduced active drug concentrations, and enhanced tumour cell survival [101]. Investigations of the regulatory role for miRNAs in impacting chemoresistance to chemotherapy agents are abundant, with several miRNA expression profiles implicated in predicting chemoresistance: Within TNBC, translational research studies have recently correlated decreased expression of miR-18a, miR-1207-5p and miR-5195-3p are predictors of resistance to paclitaxel or docetaxel in TNBC [102,103,104]. Furthermore, Wu et al. identified that overexpression of miR-620 facilitates tumour resistance to gemcitabine-based chemotherapies in TNBC through downregulating dCMP deaminase (DCTD) expression [105]. In the circulation, detection of increased miR-125b expression levels correlated with chemoresistance in 56 patients with invasive ductal carcinoma being treated with curative intent (*p* = 0.008) [106]. MiR-24 has been shown to induce chemoresistance in early breast cancer through hampering the chemotherapy-induced apoptosis and increasing cell resistance to hypoxia via the hypoxia-inducible factor-1 (HIF-1) pathway [107]. Furthermore, miR-155 has been implicated in several studies as a player in drug resistance and cancer promotion through regulation of FOXO3a signaling, interrupting TGF-*beta* facilitating epithelial–mesenchymal transition and inducing drug resistance through RhoA signaling [108]. Additionally, miR-221 has been illustrated to promote breast cancer resistance to adriamycin via modulation of the PTEN/Akt/mTOR signaling pathway in 25 breast cancer samples [109].

## 6. MicroRNAs for Therapeutic Use in Breast Cancer

**Figure 1:**
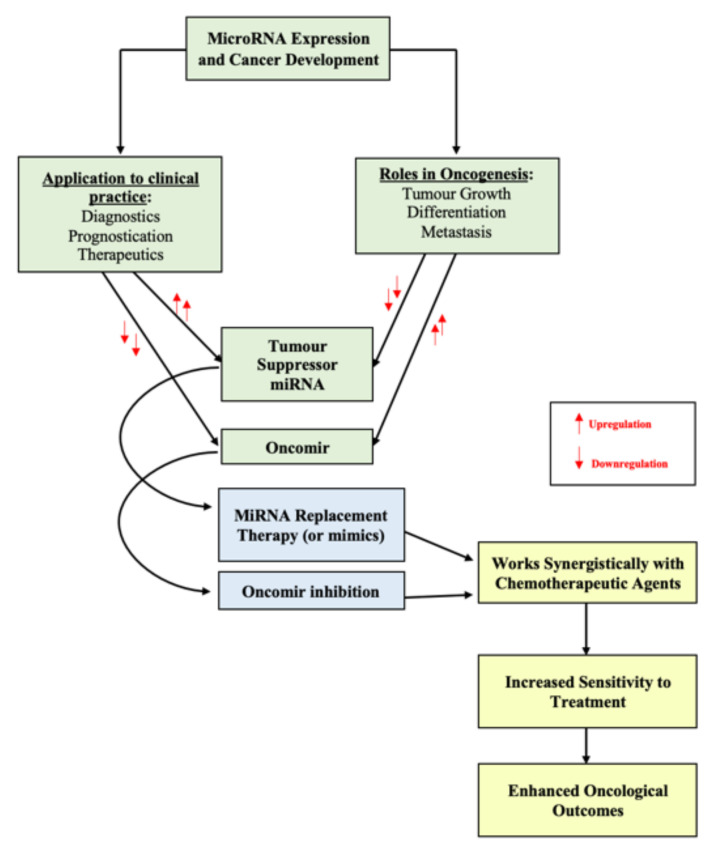
Figure outlining the manipulation of miRNA expression profiles for cancer therapeutics.

The molecular era has facilitated the use of miRNAs for the development of novel therapeutic strategies. These involve the introduction of pre-selected miRNAs into the tumour microenvironment for use as a treatment or to enhance the effect of current treatment modalities used in routine clinical practice, such as systemic chemotherapy [101,110]. MiRNAs have the capacity to function as either oncomirs or tumour suppressors, indicating there are two potential approaches for using miRNA as therapeutics—(1) oncomir inhibition which involves reducing targeted miRNA expression profiles (i.e., miRNA silencing) through introducing inhibitory miRNA to reduce the anticipated protein expression levels or (2) miRNA replacement therapy which involves inducing and overexpressing of select miRNA to reduce oncogenesis or increase sensitivity to systemic treatment (Figure 1).

### 6.1. Oncomir Inhibition

Oncomirs are classically upregulated in malignancy [111]. The inhibition of oncogenic miRNA activity may be achieved through the use of miRNA antagonist oligonucleotides (anti-miRs), targeted miRNA silencing (antagomirs), or locked nucleic acid (LNA) [112]. Such inhibitor mechanisms can augment the sensitivity of breast cancer cells to chemotherapeutic agents in several pre-clinical studies: Li et al. report the successful transfection of miR-3609 into MCF-7/ADR cell lines to increase tumour cell sensitivity to adriamycin-based chemotherapy [113]. Furthermore, Lin et al. induced miR-133 into cisplatin-resistant TNBC cells from 65 breast cancer patients and successfully increased cell sensitivity to chemotherapy for these patients [114]. Similarly, Li et al. transfected miR-155-5p into tumour cells and successfully overcame paclitaxel resistance in previously resistant breast cancer cells [115]. Finally, Mei et al. indicate that downregulation of miR-21 increased MCF-7 breast cancer cells lines to docetaxel chemotherapy [116], while Ru et al. outline how miR-203 knockdown can successfully increase cisplatin sensitivity to chemotherapy.

### 6.2. MiRNA Replacement Therapy

Tumour suppressor miRNAs have the capacity to inhibit oncogenesis through regulating oncogenes and controlling genes responsible for controlling cell proliferation and apoptosis [117]. MiRNA replacement therapy involves the reintroduction of tumour suppressing miRNA (or mimics) into the tumour microenvironment to reduce oncogenesis and control cancer proliferation [118]. Zhang et al. described the potential to use the miR-34 family as tumour suppressor modulators in the setting of several epithelial cancers, including breast [119]. The works of Yu et al. and Cochrane et al. provide data illustrating the value of reintroducing and increasing expression levels of let-7a, miR-30, and miR-200c to reduce tumourigenesis and increase chemosensitivity in studies involving animals and MDA-MB-231 and MDA-MB-549 chemoresistant breast cancer cells lines [120,121,122]. Furthermore, Kalinowski reviewed the strong therapeutic potential of miR-7 replacement therapy to enhance current treatment with conventional breast cancer chemotherapy [123].

The great challenge in current and future strategies for improved outcome in breast cancer is to successfully implement an evidence-based approach which fundamentally can allow (1) using miRNAs to address treatment rationalisation—selection of appropriate length and constituents for enhancing chemotherapeutic effect, (2) enhancing liquid biopsies selection of appropriate systemic miRNA profiling to reduce the need for cytotoxic chemotherapy/address recurrence risk, (3) augmenting current molecular subtyping with subtype-specific rational miRNA profiling, and (4) using miRNAs to enhance/select chemotherapeutic and other tumour-targeting strategies.

## 7. Future Directions for miRNA

Despite considerable investment into the discovery, development, and augmentation of miRNAs as novel therapeutics for breast cancer patient management, this subcategory of translational research remains in its infancy. Furthermore, the efforts to use miRNA to personalise cancer therapeutics have been plentiful, with minimal advancements towards enhancing clinico-oncological outcomes through miRNA targeting. Currently, the evolution of miRNA therapeutics faces several developmental challenges. This review is limited in that most studies conducted to date provide data in relation to in vitro studies, with few stemming beyond breast cancer cell lines or animal studies. In conjunction with the accepted scientific method, clinical trials evaluating the clinical efficacy, safety profiles, and cost-effective benefit are required to support the preliminary data presented by these current studies. As has been thoroughly outlined in the current review, research in the clinical trial setting has revolutionised breast cancer patient care over the past four decades, leading to novel, personalised therapeutic strategies, minimally invasive surgical approaches to the breast and axilla, and enhanced clinico-oncological outcomes for patients who in previous eras may have succumbed to their disease. With the ongoing trials evaluating novel targeted therapies such as immune checkpoint modulation [65,124] and the adoption of poly(adenosine diphosphate–ribose) polymerase inhibitors (or PARP inhibitors) into the treatment of early stage breast cancer in BRCA mutation carriers [125], the personalisation of breast cancer patient care seems even closer than ever. Thus, this review highlights the critical emphasis which must be placed on clinical trial and translational research in order to further strive towards ‘curing’ breast cancer, through the mantra of precision oncology.

## Figures and Tables

**Table 1:** Table outlining the landmark randomised clinical trials outlining the role of trastuzumab in human epidermal growth factor receptor-2 positive breast cancer.

Author	Year	Trial	N	Patients	Arms	Findings	Journal
Pegram [39]	1998	Phase II Clinical Trial	39	Refractory metastatic HER2+ breast cancer	IV trastuzumab combined with Cisplatin	Of 37 patients followed up, 24.3% (9/37) achieved a clinical PR and had SD, respectively, while 51.3% (19/37) suffered PD. Grade III/IV toxicity was observed in 56.4% (22/39)	Journal of Clinical Oncology
Cobleigh [38]	1999	Phase II Clinical Trial	213	Refractory metastatic HER2+ breast cancer	IV trastuzumab	8 patients achieved a CR (3.8%), 26 achieved a PR (12.2%), while 16 achieved an objective response (7.6%)	Journal of Clinical Oncology
Baselga [43]	1999	Phase II Clinical Trial	46	Metastatic HER2+	IV trastuzumab	Of 42 followed patients, 5 patients achieved an OR (11.6%, 5/42), specifically, 1 CR and 4 PR.	Seminars in Oncology
Slamon [37]	2001	Phase III RCT, (PIVOTAL Trial)	469	Metastatic HER2+ breast cancer	AC vs. trastuzumab combined with AC	Combined trastuzumab and chemotherapy were associated with longer PFS (7.4 months vs. 4.6 months), a higher OR rate (50% vs. 32%), a longer duration of response (9.1 months vs. 6.1 months), a lower mortality rate at 12 months (22% vs. 33%) and prolonged survival (25.1 months vs. 20.3 months) (all *p* < 0.05).	New England Journal of Medicine
Piccart-Gebhart [40]	2005	HERceptin Adjuvant (HERA) Phase III RCT (NCT00045032)	5081	Resected HER2+ breast cancer	trastuzumab alone for 2 years, trastuzumab combined with (neo)adjuvant chemotherapy for 1-year, 2 years.	Overall, there were 347 events (i.e.: recurrence, contralateral cancer, new primary, or death) of which 220 were in the observational group, compared with 127 in the trastuzumab group (HR:0.54). Cardiotoxicity was reported in 0.5% of patients treated with trastuzumab	New England Journal of Medicine
Romond [44]	2005	Phase III RCT, NSABP B-031 & N9831 (NCT00004067 & NCT00005970)	3351	Operable HER2+ breast cancer	AC and Paclitaxel vs. trastuzumab combined with AC and Paclitaxel	Overall, there were 394 events (i.e.: recurrence, new primary, or death) of which 261 were in the observational group, compared with 133 in the trastuzumab group. At 3 years, the trastuzumab group had a 12% absolute improvement in DFS and a 33% reduction in mortality.	New England Journal of Medicine
Joensuu [45]	2006	Phase III RCT—FinHer trial (ISRCTN76560285)	232	Locally advanced HER2+ breast cancer	Docetaxel or Vinorelbine, followed by FEC randomised to receive 9 trastuzumab infusions	In those treated with trastuzumab, they had enhanced 3-year RFS (HR: 0.42, 89% vs. 78%).	New England Journal of Medicine
Untch [46]	2010	GeparQuattro Phase III RCT (NCT00288002)	1509	Operable or locally advanced HER2+ breast cancer	Neoadjuvant EC-T(X) with trastuzumab	pCR rates were 31.7% in those treated for HER2+ cancers, compared with 15.7% in other subtypes. Patients with no response following EC showed an unexpectedly high pCR rate following trastuzumab (16.6% vs. 3.3% in the reference group). Cardiac toxicity was comparable for both groups.	Journal of Clinical Oncology
Slamon [34]	2011	Phase III RCT (NCT00021255)	3222	Early stage HER2+ breast cancer	AC-T vs. AC-T with trastuzumab, vs. TCH	5-year DFS rates were 75%, 84%, and 81%, with respective estimated survival rates of 87%, 92%, and 91%. The rates of cardiac dysfunction were significantly higher in the AC-T and trastuzumab group vs. TCH (*p* < 0.001).	New England Journal of Medicine
Baselga [47]	2012	NeoALLTO Phase III RCT (NCT00553358)	455	Early stage HER2+ breast cancer	Neoadjuvant lapatinib, trastuzumab, or combined lapatinib and trastuzumab	pCR rates were highest in the lapatinib and trastuzumab group (51.3%) vs. 29.5% and 24.7% in the trastuzumab and lapatinib groups, respectively. There were no major cardiac dysfunctions suffered.	Lancet
Perez [48]	2014	Phase III RCT, NSABP B-031 & N9831 (NCT00004067 & NCT00005970)	4046	Operable HER2+ breast cancer	AC and Paclitaxel vs. trastuzumab combined with AC and Paclitaxel	Adding trastuzumab to chemotherapy enhanced survival (HR: 0.63), increasing the 10-year survival from 75.2% to 84.0%. Moreover, this enhanced DFS by 40% (HR: 0.40) and improved the estimated 10-year DFS from 62.2% to 73.7%.	Journal of Clinical Oncology
Gianni [49]	2014	NeOAdjuvant Herceptin (NOAH) Phase III RCT (ISRCTN86043495)	235	Operable HER2+ breast cancer	NAC vs. NAC and trastuzumab, both received adjuvant trastuzumab	After 5 years of follow-up, patients treated with NAC and trastuzumab had an EFS of 58% vs. 43% in the NAC group (HR: 0.64). Of patients achieving a pCR (N = 67), 44 had received NAC and trastuzumab (66%) vs. 23 in those treated with NAC alone (34%).	Lancet Oncology
Cameron [50]	2017	HERceptin Adjuvant (HERA) Phase III RCT (NCT00045032)	5102	Early stage HER2+ breast cancer	Post-treatment (i.e.: surgery, (neo)adjuvant chemotherapy): trastuzumab alone for 1-year vs. trastuzumab alone for 2 years, vs. observation group	Following 11 years follow up, 1-year of trastuzumab enhanced DFS (HR: 0.76) and death (HR: 0.74) vs. observation. Receiving trastuzumab for 2 years did not improve survival vs. 1-year of treatment (HR: 1.02). Estimations of survival were 69% for 1-year, 69% for 2 years, and 63% for observations. There were increased cardiac toxicities in those treated with trastuzumab (1-year rate 4.4% and 2-year rate of 7.3%) vs. observations (0.9%)	New England Journal of Medicine
Earl [51]	2019	PERSEPHONE Phase III RCT (NCT00712140)	4089	Early stage HER2+ breast cancer	Post-treatment (i.e.: surgery, (neo)adjuvant chemotherapy): Adjuvant trastuzumab for 1-year vs. trastuzumab for 6 months	At 5 years follow up, treatment with 6-month of trastuzumab in the adjuvant setting is non-inferior to 12-month treatment after conventional treatment. Events were comparable for both groups (1-year: 12% vs. 6 months: 13%), with 4-year DFS rates of 89.4% and 89.8%, respectively (HR: 1.07). There were fewer toxicities reported in the 6-month group (19% vs. 24%)	New England Journal of Medicine

N; number, HER2+; human epidermal growth factor receptor-2 positive, IV; intravenous, PR; partial response, SD; stable disease, PD; progressive disease, OR; objective response, CR; complete response, RCT; randomised controlled trial, PFS; progression-free survival, RFS; recurrence-free survival, EFS; event-free survival, pCR: pathological complete response, NAC; neoadjuvant chemotherapy, AC; Doxorubicin and Cyclophosphamide, EC-T(X); Doxorubicin and Cyclophosphamide followed by Docetaxel with or without Capecitabine, TTP; time-to-progression, FEC: 5-flurouracil, epirubicin and cyclophosphamide, AC-T; doxorubicin and cyclophosphamide, followed by docetaxel, TCH; trastuzumab, docetaxel, and carboplatin.

**Table 2:** The outline of seminal studies validating neoadjuvant chemotherapy prescription in breast cancer patients.

Author	Year	Study	N	Patients	Arms	Findings	Journal
Fisher [58]	1998	NSABP B-018 phase III, RCT	1523	Locally advanced breast cancer	Neoadjuvant vs. adjuvant chemotherapy prescription	Overall, 13% achieved a pCR to NAC, 36% achieved a CCR, 43% achieved a PCR, and 37% of patients downstaged their axilla previously palpable LNs. Overall, patients after NAC were more likely to undergo successful BCS (67% vs. 60%, *p* = 0.002)	Journal of Clinical Oncology
Mauri [68]	2005	Meta-analysis of RCTs	3946	Early breast cancer	NAC vs. adjuvant chemotherapy	There was no difference in DP (RR: 0.99), DR (RR: 0.94), or OS (RR: 1.00) outcomes for NAC vs. adjuvant therapy. However, there were increased LRR rates following NAC (RR: 1.22)	Journal of the National Cancer Institute
Bear [61]	2006	NSABP B-027 phase III, RCT	2411	Early breast cancer	NAC (AC) and Docetaxel vs. AC alone	There were increased pCR rates and axillary downstaging with added neoadjuvant docetaxel, however failed to increase BCS rates, DFS, and OS outcomes overall. The addition of neoadjuvant Docetaxel increased pCR rates	Journal of Clinical Oncology
Van Nes [69]	2009	Preoperative chemotherapy in Primary Operable Breast Cancer (POCOB)	698	Early breast cancer	NAC vs. adjuvant chemotherapy	At 10 years of follow-up, there was no observed difference in OS, DFS, or LRR (all P>0.05); however, NAC was associated with increased BCS rates	Breast Cancer Research and Treatment
EBCTCG [55]	2018	Meta-analysis of RCTs	4756	Early breast cancer	NAC vs. adjuvant chemotherapy	At 15 years follow-up, NAC was associated with increased LRR rates (21.4% vs. 15.9%), however there was no difference in DR (38.2% vs. 38.0%), BCM (34.4% vs. 33.7%) and OS (40.9% vs. 41.2%)	Lancet Oncology

N; number, RCT; randomised controlled trial, pCR: pathological complete response, NAC; CCR; complete clinical response, PCR; partial clinical response, LN; lymph nodes, BCS; breast conservation surgery, DP; disease progression, RR; rate ratio, DR; distant recurrence, OS; overall survival, DFS; disease-free survival, LRR; locoregional recurrence, AC; Doxorubicin and Cyclophosphamide, EBCTCG; early breast cancer triallist collaborative group, BCM; breast cancer mortality.

**Table 3:** Table illustrating prospective trials evaluating the role of miRNAs in predicting response to neoadjuvant therapies.

Author	Year	Study	N	Patients	Treatment Arms	Findings	Journal
Muller [90]	2014	Prospective phase II Geparquinto Trial (NCT00567554)	127	Early stage HER2+ breast cancer	NAC with trastuzumab or lapatinib	Increased miR-21, miR-210, and miR-373 in patient’s serum following treatment with NAC correlated to response to treatment.	Breast Cancer Research and Treatment
Xue [91]	2015	Prospective phase II clinical trial	50	Early stage breast cancer	Carboplatin and Paclitaxel	Increased miR-621 expression profiles predicted pCR to NAC	Oncogene
Stevic [87]	2018	Prospective phase II clinical trial GeparSixto Trial (NCT01426880)	211	Early stage breast cancer	Docetaxel or Paclitaxel +/− Carboplatin	Aberrant miR-199a expression correlates to pCR following neoadjuvant therapies	BMC Cancer
Zhu [92]	2018	Prospective phase II clinical trial (NCT02041338)	24	Operable breast cancer	Epirubicin & Docetaxel	After the second cycle of NAC, reduced miR-34a expression was correlated with patients who did not respond to treatment	Cancer Medicine
Kahraman [93]	2018	Prospective, case–control study (MODE-B study)	42	Early stage TNBC breast cancer	Carboplatin and Paclitaxel	Identification of 74 miRNAs which predicted pCR based on changes in expression profiles pre- and post-NAC.	Scientific Reports
Di Cosimo [80]	2019	NeoALLTO Phase III RCT (NCT00553358)	455	Early stage HER2+ breast cancer	Neoadjuvant lapatinib, trastuzumab, or combined lapatinib and trastuzumab	Increased circulating plasma levels of miR-140a-5p, miR-148a-3p and 374a-5p were associated with pCR and miR-140a-5p predicted enhanced EFS	Clinical Cancer Research
Lindholm [94]	2019	Randomised, phase II clinical trial (NCT00773695)	132	Early stage HER2- breast cancer	FEC-T or FEC-P, +/− Bevacizumab	Hierarchical clustering of 627 miRNAs with response at 12 and 25 weeks to neoadjuvant treatment with NAC or NAC combined with Bevacizumab; of these, 217 had differential expression profiles (71 upregulated and 146 downregulated) between responders and non-responders.	Molecular Oncology
Rodriguez-Martinez [95]	2019	Prospective clinical trial	53	Locally advanced and advanced breast cancer	AC	Exosomal expression of miR-21 correlated in a stepwise fashion with patients achieving a CR having significantly reduced miR-21 vs. patients with PR and SD, respectively.	Breast Cancer Research
Di Cosimo [86]	2020	NeoALLTO Phase III RCT (NCT00553358)	455	Early stage HER2+ breast cancer	Neoadjuvant lapatinib, trastuzumab, or combined lapatinib and trastuzumab	After 2 weeks of neoadjuvant treatment, increased expression of miR-15a-5p, miR-140-3p, miR-320a, miR-320b, miR-363-3p, miR-378a-3p, miR-486-5p & miR-660-5p and decreased miR-30d-5p correlated with pCR to lapatinib. At 2 weeks of therapy, increased expression of miR-26a-5p & miR-374b-5p correlated with pCR to trastuzumab. Increased let-7g-5p & miR-191-5p and reduced miR-195-5p correlated with pCR to combined trastuzumab and lapatinib.	International Journal of Molecular Sciences
McGuire [79]	2020	Prospective phase II clinical trial [CTRIAL-IE ICORG] 10/11 (NCT00553358	114	Early stage breast cancer	Various NAC regimens	Responders had reduced miR-21 and miR-195 vs. non-responders in all breast cancer subtypes. MiR-21 independent predicted response (OR 0.538, 95% CI 0.308–0.943). In luminal cancers, reduced expression of miR-145 and miR-21 correlated with response to NAC.	Cancers (Basel)
Zhang [96]	2020	Prospective phase II trials; SHPD001 (NCT02199418) & SHPH02 (NCT02221999)	65	Early stage HER2+ breast cancer	Paclitaxel, Cisplatin & trastuzumab	Low miR-222-3p expression was predictive of achieving pCR (OR: 0.258, 95% confidence interval: 0.070–0.958, *p* = 0.043) and favourable DFS and survival	Frontiers in Oncology

N; number, HER2-+ human epidermal growth factor receptor-2 positive NAC; neoadjuvant chemotherapy, pCR; pathological complete response, TNBC; triple-negative breast cancer, EFS; event-free survival, HER2-; human epidermal growth factor receptor-2 negative, FEC-T; 5-fluorouracil, epirubicin, and cyclophosphamide followed by docetaxel, FEC-P; 5-fluorouracil, epirubicin, and cyclophosphamide followed by paclitaxel, AC; doxorubicin and cyclophosphamide, CR; complete response, PR: partial response, SD; stable disease, OR; odds ratio, DFS; disease-free survival.

## Data Availability

Not applicable.

## Supplementary Materials

The following are available online at https://www.mdpi.com/article/10.3390/ijms221910812/s1.
