# Supplementary material for: MicroRNA Expression Profiles and Breast Cancer Chemotherapy
Source: Int J Mol Sci. 2021 Oct 6;22(19):10812. doi: 10.3390/ijms221910812 (PMC8509379; doi:10.3390/ijms221910812)

MicroRNA Expression Profiles and Breast Cancer Chemotherapy

Matthew G. Davey MCh MRCS, Aoife J. Lowery PhD FRCS,

Nicola Miller PhD, Michael J. Kerin MCh FRCS FRCSI FRCSEd

Department of Surgery, The Lambe Institute for Translational Research, National University of Ireland, Galway, Galway, Republic of Ireland, H91 YR71

Supplementary

Supplementary

Table S1 outlining the landmark clinical studies and trials including multigene expression assays for guiding adjuvant chemotherapy prescription in early stage estrogen receptor positive breast cancer.

Author	Year	Multigene Assay	Study Type	N	Technique	Findings	Journal
van de Vijver [107]	2002	MP 70-gene expression assay	RC	295	Microarray expression profiling	In stage I/II breast cancer patients aged 51 years or less (144 LN+, 151 LN-), MP successfully substratified these patients: 10-year survival outcomes for classed to have GHR was 54.6% versus 94.5% for those classes to have a GLR	New England Journal of Medicine
Paik [9]	2004	RS 21-gene assay (Genomic Health Inc.)	RC	668	Real-time polymerase chain reaction on paraffin-embedded blocks	RS successfully predicted risk of breast cancer recurrence in LN-ER+/HER2-: 6.8% recurrence risk in low-risk group (RS<18), 14.3% recurrence risk in intermediate-risk group (RS 18-30), and 30.5% recurrence risk in high-risk group (RS >30) at 10-year follow-up	New England Journal of Medicine
Buyse [108]	2006	MP 70-gene expression assay	TRANSBIG	307	Microarray expression profiling	At 13.6 years follow up, the GLR had favourable DMFS and OS compared to the GHR (10-year OS: 89% vs. 69%, DMFS: HR: 2.32, OS: 2.79)	Journal of the National Cancer Institute
Parker [109]	2009	PAM50 gene expression assay	RC	1,083	Microarray expression profiling and Real-time polymerase chain reaction	PAM50 successfully classified breast cancer based on intrinsic molecular subtypes which outline the benefit from chemotherapy prescription and likelihood of achieving pCR to NAC.	Journal of Clinical Oncology
Mook [110]	2009	MP 70-gene expression assay	RC	241	Microarray expression profiling	MP outlined the 10-year DMFS and BCSS for the GLR was 91% and 94%, versus 76% and 76% respectively for the GLR	Breast Cancer Research and Treatment
Albain [111]	2010	RS 21-gene assay (Genomic Health Inc.)	SWOG 8814	367	Real-time polymerase chain reaction	RS successfully provided prognostication for tamoxifen treated patients with LN+ ER+/HER2- breast cancer: Those with RS<18 could be spared CET while those with RS>30 derived major benefit from receiving chemotherapy.	Lancet Oncology
Nielsen [112]	2010	PAM50 gene expression assay	RC	786	Microarray expression profiling and Real-time polymerase chain reaction	PAM50 projected prognoses for molecular subtypes based on predicted survival for those classed as having luminal A disease; luminal B (HR: 1.99), HER2 enriched (HR: 3.65) and TNBC (HR; 17.71).	Clinical Cancer Research
Filiptis [113]	2011	EP 12-gene expression assay	RC using tumour blocks from two phase III clinical trials: NCT00309491 NCT00291759	1,702	Real-time polymerase chain reaction	Based on results of the two trials, EP predicted 10-year ROR as 4% and 4% for the low risk group and 28% and 22% respectively for the high risk patients from each trial.	Clinical Cancer Research
Drukker [114]	2013	MP 70-gene expression assay	MicroarRAy-prognoSTics-in-breast-cancER study (or RASTER) (ISRCTN719179 16)	427	Microarray expression profiling	Patients with cT1-3/N0 disease were enrolled: Those with GLR (N=219) had 97.0% DRFI versus 91.0% in GHR (N=91.7%). In GLR, 124 patients were classed as 'high-risk' based on clinicopathological data – 76.0% of these were spared adjuvant systemic treatment and overall, 5-year DRFI were 98.0% for GLR.	International Journal of Cancer

Dubsky [10]	2013	EP 12-gene expression assay	RC using tumour blocks from two phase III clinical trials: NCT00309491 NCT00291759	1,702	Real-time polymerase chain reaction	EP substratified ER+/HER2- cancer into two clinical distinct subtypes: 64% of cases were classed as having a 1.8% risk of DM at 10-years follow up.	British Journal of Cancer
Saghatchian [115]	2013	MP 70-gene expression assay	TRANSBIG	173	Microarray expression profiling	In patients with ER+/HER2-/LN4-9 disease, 5-year DMFS was 87% for GLR versus 63% in those with GHR. OS for GLR was 97% versus 63% for GHR.	Breast
Petkov [116]	2016	RS 21-gene assay (Genomic Health Inc.)	RC	43,259	Real-time polymerase chain reaction	In patients with ER+/HER2-/LN- disease (N=38,568), 5-year BCSM were 0.4%, 1.4% and 4.4% for RS<18, RS 18-30 and RS>30. For those with LN+ (micrometastases or LN1-3, N=4,691) disease 5-year BCSM were 1,0%, 2.3% and 14.3% respectively.	npj Breast Cancer
Stein [117]	2016	PAM50 gene expression assay	OPTImal personalised treatment for early breast cancer using Multi-parameter Analysis (or OPTIMA) (ISRCTN42400492)	313	Microarray expression profiling and Real-time polymerase chain reaction	Trial ongoing: Recruited and randomised 313 patients to CET or chemotherapy for RS>25 and CET or ET for RS<25. Included patients have ER+/HER2-/LN1-9 or tumour size greater than 30mm.	Health Technology Assessment
Nitz [118]	2017	RS 21-gene assay (Genomic Health Inc.)	West Germany Study Group (WSG)PlanB trial NCT01049425	32,247	Real-time polymerase chain reaction	Patients with ER+/HER2-/LN- or LN1-3 substratified using RS into RS<11 treated with ET had comparable DFS and OS to those with RS 11-25 and RS>25 treated with CET at 5-years follow up (DFS: 94%, 94% and 84%, OS: 99%, 97% and 93% respectively).	Breast Cancer Research and Treatment
Sparano [25]	2018	RS 21-gene assay (Genomic Health Inc.)	Trial Assigning IndividuaLized Options for Treatment (Rx) (or TAILORx) (NCT00310180)	10,273	Real-time polymerase chain reaction	Adjuvant ET was non-inferior to combined CET in patients diagnosed with LN- ER+/HER2- disease with RS 11-25. CET was beneficial in those aged 50-years or less or in premenopausal patients.	New England Journal of Medicine
Laenkholm [119]	2018	PAM50 gene expression assay	RC	2,558	Microarray expression profiling and Real-time polymerase chain reaction	In ER+/HER2-/LN+ cases, 26% of cases were classed as low ROR (DR risk 3.5%) versus a DR risk of 22.1% for those classed high ROR. In ER+/HER2-/LN- patients, those classed low ROR had a DR risk of 5.0% versus 17.8% in high ROR group.	Journal of Clinical Oncology
Tsai [120]	2018	MP 70-gene expression assay	PROspective study of Mammaprint in breast cancer patients with Intermediate recurrence Score (or PROMIS) (NCT01617954)	840	Microarray expression profiling	In 840 patients with RS 18-30, MP provided actionable information with a change in treatment decision (i.e.: CET vs ET) in 282 of these patients (33.6%) (OR: 0.64). In GLR patients, 108/374 had chemotherapy removed; in GHR patients, 171/446 had chemotherapy removed.	JAMA Oncology

Filiptis [121]	2019	EP 12-gene expression assay	RC using tumour blocks from two phase III clinical trials: NCT00309491 NCT00291759	1,702	Real-time polymerase chain reaction	Based on follow up results of the two trials, 10-year DRFS was 95.5% for GLR patients versus 80.9% for GHR patients. For those with LN-, DRFS for GLR was 95.5% versus 87.0%. In cases of LN1-3, DRFS was 95.6% for GLR versus 80.9% for GHR patients at 10-years follow up.	Clinical Cancer Research
Cordosa [122]	2020	MP 70-gene expression assay	Microarray In Node negative Disease may Avoid ChemoTherapy (or MINDACT) (NCT00433589)	6693	Microarray expression profiling	MP outlined real world data illustrating 8-year DMFS and OS for the 'clinical high-risk, GLR patients was 92% and 95.7% with CET and 89.4% and 94.3% respectively.	Journal of Clinical Oncology
Kalinsky [29]	2021	RS 21-gene assay (Genomic Health Inc.)	SWOG S1007: Rx for POsitive NoDes, Endocrine Responsive breast cancer (or RxPONDER) (NCT01272037)	9,383	Real-time polymerase chain reaction	Postmenopausal patients with ER+/HER2- LN1-3 disease may be de-escalated from CET to ET alone, however there is iDFS benefit for those treated with CET in premenopausal patients (5-year iDFS: 94.2% vs. 89.0%)	Cancer Research (proceedings from the 2020 San Antonio Breast Cancer Virtual Symposium, San Antonia, Texas, USA)

N; number, RS; MP; MammaPrint 70-gene expression signature, GLR; genomic low-risk, GHR; genomic low-risk, OncotypeDX Recurrence Score©, EP; Endopredict® 12-gene expression assay, RC: retrospective cohort, LN-; lymph node negative, LN+; lymph node positive, ER+; estrogen receptor positive, PAM50; Prosigna©, Predictor Analysis of MicroArray 50, HER2-; human epidermal growth factor receptor-2 negative, ET; endocrine therapy, CET; chemoendocrine therapy, LN1-3; one to three positive lymph nodes, LN1-4; four to nine positive lymph nodes, LN1-9; one to nine positive lymph nodes, ROR; risk of recurrence, DR; distant recurrence, pCR; pathological complete response, NAC; neoadjuvant chemotherapy, iDFS; invasive disease-free survival, DMFS; distant metastasis-free survival, BCSS; breast cancer specific survival, BCSM; breast cancer-specific mortality, DRFI; distant recurrence-free interval, DRFS; distant recurrence-free survival, HR; hazards ratio, OR; odds ratio, TNBC; triple negative breast cancer.

Figure S1 illustrating the current multimodal therapeutic strategies for breast cancer patient management.

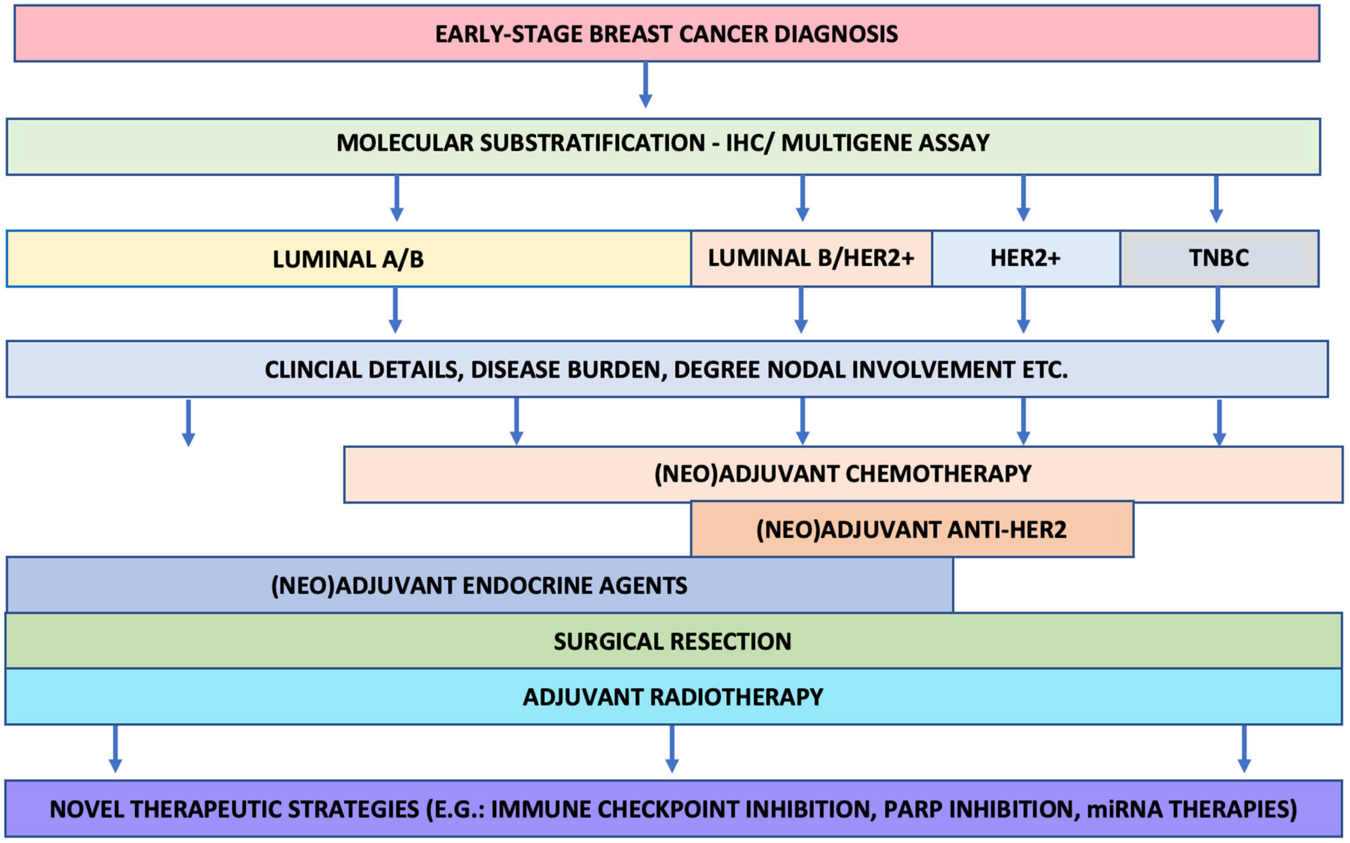

Supplement: Supplementary file 1 [file ijms-22-10812-s001.zip › ijms-1395289-supplementary.pdf]
